# Integrative Review of Molecular, Metabolic, and Environmental Factors in Spina Bifida and Congenital Diaphragmatic Hernia: Insights into Mechanisms and Emerging Therapeutics

**DOI:** 10.3390/cells14141059

**Published:** 2025-07-10

**Authors:** Angelika Buczyńska, Iwona Sidorkiewicz, Przemysław Kosiński, Adam Jacek Krętowski, Monika Zbucka-Krętowska

**Affiliations:** 1 Clinical Research Centre, Medical University of Białystok, ul. M. Skłodowskiej-Curie 24a, 15-276 Białystok, Poland; adamkretowski@wp.pl; 2 Clinical Research Support Centre, Medical University of Białystok, ul. M. Waszyngtona 17, 15-276 Białystok, Poland; iwona.sidorkiewicz@umb.edu.pl; 3 Department of Obstetrics, Perinatology and Gynecology, Medical University of Warsaw, 63A Zwirki i Wigury, 02-091 Warsaw, Poland; przemyslaw.kosinski@wum.edu.pl; 4 Department of Endocrinology, Diabetology and Internal Medicine, Medical University of Białystok, ul. Sklodowskiej-Curie 24a, 15-276 Białystok, Poland; 5 Department of Gynecological Endocrinology and Adolescent Gynecology, Medical University of Białystok, ul. M. Skłodowskiej-Curie 24a, 15-276 Białystok, Poland

**Keywords:** Spinal Bifida, Diaphragmatic Hernia, genetic pattern, metabolic markers

## Abstract

Spina Bifida (SB) and Congenital Diaphragmatic Hernia (CDH) are complex congenital anomalies that pose significant challenges in pediatric healthcare. This review synthesizes recent advancements in understanding the genetic, metabolic, and environmental factors contributing to these conditions, with the aim of integrating mechanistic insights into therapeutic innovations. In SB, key findings highlight the roles of *KCND3*, a critical regulator of spinal cord development, and *VANGL2*, essential for planar cell polarity and neural tube closure. MicroRNAs such as miR-765 and miR-142-3p are identified as key regulators of these genes, influencing neural development. Additionally, telomere shortening—a marker of cellular senescence—alongside disruptions in folate metabolism and maternal nutritional deficiencies, significantly increases the risk of SB. These findings underscore the crucial role of telomere integrity in maintaining neural tissue homeostasis during embryonic development. For CDH, genetic deletions, including those on chromosome 15q26, and chromosomal abnormalities have been shown to disrupt lung and vascular development, profoundly impacting neonatal outcomes. MicroRNAs miR-379-5p and miR-889-3p are implicated in targeting essential genes such as *IGF1* and *FGFR2*, which play pivotal roles in pulmonary function. Promising emerging therapies, including degradable tracheal plugs and fibroblast growth factor-based treatments, offer potential strategies for mitigating pulmonary hypoplasia and improving clinical outcomes. This review underscores the intricate interplay of genetic, metabolic, and environmental pathways in SB and CDH, identifying critical molecular targets for diagnostics and therapeutic intervention. By integrating findings from genetic profiling, in vitro models, and clinical studies, it aims to inform future research directions and optimize patient outcomes through collaborative, multidisciplinary approaches.

## 1. Introduction

Spina Bifida (SB) and Congenital Diaphragmatic Hernia (CDH) are congenital anomalies that carry profound implications for affected individuals, presenting significant challenges in pediatric healthcare. SB occurs in approximately 1 in 1000 live births globally, whereas CDH has an estimated incidence ranging from 1 in 2500 to 1 in 5000 live births, with notable regional variability influenced by genetic and environmental factors [1,2,3,4]. These conditions are influenced by complex genetic, environmental, and developmental factors [3,5,6]. Despite advancements in prenatal screening and management, the complex and multifactorial nature of SB and CDH, along with their variable clinical presentations, continues to present significant challenges in pediatric care. Recent research has increasingly focused on elucidating the genetic and molecular mechanisms underlying SB and CDH, with the goal of improving understanding, diagnosis, and treatment strategies [7,8]. Investigating the role of metabolic factors in the pathogenesis of SB and CDH is critical for advancing diagnostic and therapeutic strategies, as well as for unraveling the intricate processes involved in fetal development [9,10,11,12,13].

Although SB and CDH are traditionally considered distinct anomalies arising from different germ layers and embryological stages, recent evidence suggests partial overlap in the molecular and environmental pathways that govern early fetal development. Specifically, shared disruptions in folate metabolism, oxidative stress response, and microRNA regulation may impact both neural tube closure and diaphragm formation. However, the rarity of their co-occurrence in clinical practice is likely due to their divergence in tissue origin—neuroectodermal in SB versus mesodermal/neural crest in CDH—and critical windows of vulnerability. By exploring these shared and distinct molecular mechanisms, this review aims to elucidate why such dual presentations are infrequent yet biologically plausible, highlighting opportunities for cross-condition diagnostic insights.

Both conditions result from early disruptions in embryonic development and are influenced by a multifactorial etiology, including genetic mutations, epigenetic alterations, oxidative stress, and nutritional deficiencies such as folate metabolism disturbances [5,10,12,13,14,15]. Recent evidence highlights shared molecular signatures, including the involvement of microRNAs (miRNA) (e.g., miR-379-5p, miR-142-3p) and telomere shortening, which have been implicated in both SB and CDH pathogenesis as markers of genomic instability and impaired cellular homeostasis [15,16,17]. Furthermore, both anomalies are associated with chromosomal abnormalities, aneuploidies, and copy number variants (CNVs), suggesting convergent genetic vulnerability [18,19,20,21]. Emerging therapeutic strategies—such as fetal interventions (e.g., fetoscopic endoluminal tracheal occlusion (FETO) in CDH, in utero repair in SB), growth factor therapies, and gene regulation approaches—also demonstrate a shared direction of translational research [22,23,24].

Given these overlapping pathways and clinical parallels, a combined review enables the identification of shared targets for diagnosis, risk assessment, and treatment development. This integrative approach provides a broader systems-level perspective and highlights opportunities for cross-disciplinary innovations in fetal medicine.

The primary objective of this integrative review is to synthesise current knowledge on the genetic, epigenetic, metabolic, and environmental drivers of SB and CDH, identify shared developmental pathways, and highlight translational opportunities for diagnosis, risk stratification, and prenatal therapy. Thus, this review synthesized recent findings on the genetic and molecular mechanisms underlying SB and CDH, integrating insights from in vitro models and novel medical approaches. By exploring the roles of specific genes, the impact of chromosomal abnormalities, and the potential of emerging therapies, this review aims to provide a comprehensive overview of current knowledge while identifying key areas for future research in the diagnosis and treatment of these congenital anomalies.

## 2. Materials and Methods

### 2.1. Revised Material and Methods

This review constitutes a comprehensive narrative review with a semi-systematic literature selection process, aimed at synthesizing molecular, genetic, epigenetic, metabolic, and environmental factors contributing to SB and CDH, including their potential mechanistic overlap.

A structured literature search was performed using the electronic databases PubMed, Scopus, Web of Science, MEDLINE, and Google Scholar. Search terms were combined using Boolean operators and included: (“spina bifida” OR “neural tube defects” OR “NTDs”) AND (“genetic factors” OR “microRNA” OR “copy number variants” OR “folate metabolism” OR “oxidative stress”), as well as (“congenital diaphragmatic hernia” OR “CDH”) AND (“genetic mutation” OR “CNVs” OR “molecular pathway” OR “lung development” OR “retinoic acid”).

The search was limited to English-language full-text articles published between 2000 and 2024. A total of 1024 articles were retrieved. After the removal of 546 duplicates using Zotero 7.1 (Corporation for Digital Scholarship, George Mason University, Fairfax, VA, USA), 478 records underwent title and abstract screening. Of these, 318 were excluded for not meeting basic relevance criteria. The full texts of 160 articles were reviewed in detail.

Inclusion criteria:

- Original human or animal studies presenting molecular, genetic, epigenetic, or metabolic data related to SB or CDH;
- Experimental in vitro or in vivo studies focusing on developmental pathways, gene expression, microRNAs, or chromosomal alterations;
- Systematic reviews or meta-analyses with primary molecular data or gene pathway integration.

Exclusion criteria:

- Clinical case reports or reviews without molecular relevance;
- Duplicates, abstracts only, non-English publications, or studies focusing solely on surgical outcomes or late clinical course.

Final inclusion: A total of 96 studies were included in the qualitative synthesis, comprising:

- 41 human molecular studies,
- 29 animal or in vitro experimental studies,
- 26 systematic or mechanistic reviews.

To ensure quality and relevance, a narrative risk of bias assessment was applied, taking into account methodological transparency, presence of appropriate controls, clarity of molecular endpoints, and relevance to human developmental biology. Due to heterogeneity of study types, a formal quantitative bias scoring system (e.g., ROBINS-I) was not applied.

**Figure 1:**
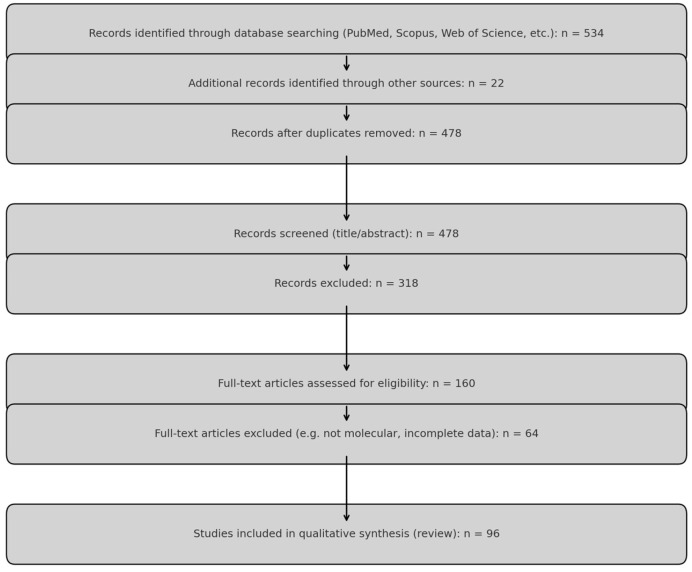
PRISMA flowchart diagram.

The literature selection process is summarized in a standardized PRISMA 2020 flow diagram (Figure 1).

### 2.2. Inclusion and Exclusion Criteria

To ensure methodological rigor and thematic relevance, studies were selected based on clearly defined inclusion and exclusion parameters. Eligible publications included original research articles and systematic reviews published in peer-reviewed journals between 2000 and 2024, focusing on molecular, genetic, epigenetic, metabolic, or environmental factors implicated in the pathogenesis of SB or CDH. Particular emphasis was placed on studies that presented clearly delineated outcomes, utilized validated experimental techniques, and offered clinically relevant insights such as the identification of biomarkers or therapeutic targets. Both in vitro and in vivo studies, as well as those involving human biological material (e.g., fetal tissue, amniotic fluid, or genomic datasets), were considered. Articles were excluded if they were non-peer-reviewed, not published in English, or lacked direct relevance to the mechanistic understanding of SB or CDH. Studies with inadequate methodological transparency or insufficient data validation were also omitted. Among the included literature, prioritization was given to those with large sample sizes, multicenter design, innovative insights into disease pathways, or application of high-throughput molecular techniques such as miRNA profiling or copy number variant analysis. Two independent reviewers applied these criteria during screening and full-text assessment, with any discrepancies resolved through discussion or consultation with a third reviewer to ensure consistency in the selection process.

### 2.3. Study Selection Process

Two independent reviewers assessed the relevance of studies by screening titles and abstracts. Full-text reviews were conducted for potentially eligible studies, and disagreements were resolved through discussion or by a third reviewer. The findings from selected studies were synthesized to identify key genetic and metabolic mechanisms and their clinical implications.

### 2.4. Quality Assessment and Scoring

The quality of included studies was evaluated using established assessment tools, including the Newcastle-Ottawa Scale for observational studies and the PRISMA checklist for systematic reviews [25,26,27]. Scores were assigned to each study based on methodological rigor, with numerical scores used to grade studies on aspects such as study design, sample size, and data validity. These scores were categorized into high, moderate, and low-quality groups, and their influence on the conclusions was weighted accordingly. Low-quality studies were not excluded but were assigned less weight in the synthesis of findings, ensuring transparency while minimizing bias.

### 2.5. Data Extraction and Synthesis

Data were extracted systematically, including study design, patient characteristics, genetic and metabolic markers assessed, and study outcomes. A narrative synthesis approach was used to integrate findings, emphasizing commonalities and disparities across studies. The synthesis aimed to elucidate the genetic and metabolic mechanisms underlying SB and CDH and to explore their potential clinical implications. Key findings were summarized and discussed in subsequent sections, offering insights into the pathways driving these anomalies and identifying potential targets for diagnostics and therapy.

## 3. Epidemiology

### 3.1. Spinal Bifida

A neural tube defect (NTD) represents a broad category of congenital abnormalities affecting the central nervous system (CNS) due to incomplete neural tube closure [28]. These conditions have a global occurrence, with an estimated incidence ranging from 1.0 to 10.0 per 1000 newborns [29]. Annually, approximately 1427 infants are delivered with SB, equating to an occurrence of 1 in 2758 live births worldwide [30]. The predominant types of NTDs primarily consist of anencephaly, characterized by incomplete closure in the cranial region, and SB, marked by deficient closure below the cranial region. SB functions as an overarching designation encapsulating diverse subcategories of congenital anomalies, including myelomeningocele (MM), meningocele, and lipomeningocele [31]. Among these subtypes, MM, characterized by the protrusion of nervous tissue and its protective coverings through a vertebral defect, stands as the most prevalent, accounting for over 90% of all SB cases. Environmental factors such as maternal folate deficiency, hyperhomocysteinemia, and poor dietary methyl donor availability are among the best-established non-genetic contributors to neural tube defects, acting through disrupted one-carbon metabolism and epigenetic instability.

### 3.2. Diaphragmatic Hernia

CDH is a malformation originating from an aberration in diaphragmatic development, resulting in the protrusion of abdominal contents into the thoracic cavity [32]. The frequency of CDH is estimated at approximately 1 to 4 cases per 10,000 live births and exhibits variability among different populations [2]. The classification of the hernias depends on the specific location of the diaphragmatic defect. Bochdalek hernias, the most prevalent type (70% to 75%), arise from defects in the postero-lateral portion of the diaphragm, predominantly located on the left side, with less frequent occurrences on the right side. Morgagni hernias, constituting 20% to 25%, result from defects in the anteromedial part of the diaphragm, while central hernias account for 2% to 5% of cases [33]. Bilateral defects are exceedingly rare and portend a grave prognosis. While certain studies have reported a marginally higher incidence among males, numerous other investigations have failed to establish a consistent correlation with gender [34] (Table 1).

## 4. Spinal Bifida

### 4.1. Etiology

SB results from the incomplete closure of the posterior spinal elements and typically manifests between the 17th and 30th days of fetal development. The process of neuralization unfolds in two distinct phases: primary and secondary neuralization [38]. Primary neuralization involves the closure of the neural tube, responsible for the formation of the brain and spinal cord. Secondary neuralization encompasses the development of the caudal structures of the neural tube, giving rise to the sacral and coccygeal regions. These caudal structures initiate their development around day 26 of gestation, and any failure in their proper closure results in varying degrees of spinal dysraphism [36]. The etiology of neural tube development defects is deemed multifactorial, influenced by a combination of environmental and genetic factors. Folate deficiency represents the most prevalent environmental cause, with a substantial proportion of cases categorized as “folic acid sensitive.” Global initiatives fortifying dietary folate have resulted in a 28% reduction in the prevalence of anencephaly and SB. However, folic acid fortification has been implemented in various countries at different stages of advancement. In developed countries, such as the United States and Canada, these policies have proven effective in reducing the incidence of SB. However, in many African and developing countries, despite the existence of guidelines, there are still challenges in ensuring widespread access to fortified foods, which is associated with higher rates of SB. The effectiveness of folic acid fortification policies largely depends on the availability of these products and the successful implementation of such measures within a given country [14].

Several environmental exposures have been shown to induce diaphragmatic and pulmonary defects in animal models, notably nitrofen and other compounds that disrupt retinoic acid signaling. Furthermore, maternal hypoxia, hyperglycemia, and vitamin A deficiency have been associated with altered mesodermal and neural crest development relevant to CDH pathogenesis. Additional environmental risk factors include maternal obesity, maternal diabetes, and teratogenic agents such as valproic acid, the latter carrying a markedly elevated tenfold risk of contributing to NTDs [35]. Certain genetic factors have also been associated with impaired neuralization, including several chromosomal syndromes and genetic polymorphisms. Notably, research has implicated the methylenetetrahydrofolate reductase (*MTHFR*) NM_005957.5:c.665C > T (C677T) gene polymorphism encoding the methylenetetrahydrofolate reductase, which plays a role in folate metabolism, as a potential genetic risk factor [39]. While NTDs are typically isolated defects, some are linked to chromosomal aneuploidies, most frequently Trisomy 13 and 18 [6,40].

### 4.2. Genetic-Based Studies

Genetic profiling in SB has emerged as a critical area of research, aiming to uncover the genetic mutations and alterations that contribute to the development and progression of this complex congenital anomaly. The study conducted by Li et al. involves the analysis of transcription profiles from human amniocytes, obtained from individuals with SB and healthy controls, which were retrieved from the Gene Expression Omnibus database. Outlier data are first removed using principal component analysis (PCA) and sample clustering. Genes in the top 25% variance from the GSE4182 dataset are then selected for analysis through Weighted Gene Co-expression Network Analysis (WGCNA). After preprocessing, 5407 genes are analyzed, resulting in nineteen distinct modules. From these, 967 candidate genes potentially involved in SB pathology are identified by integrating co-expression networks with significantly differentially expressed genes [15]. Additionally, the research incorporates previous miRNA microarray results, leading to the construction of a miRNA–mRNA network, involving four miRNAs and 39 mRNAs. Notably, three key genes are linked to two miRNA-associated gene networks. To validate the findings, quantitative real-time PCR (qRT-PCR) is employed, confirming the upregulation of potassium voltage-gated channel, Shal-related subfamily member 3 *(KCND3*) in SB. *KCND3* is linked to neural tube development in SB. A de novo *KCND3* mutation has been shown to cause severe Kv4.3 channel dysfunction, leading to early-onset cerebellar ataxia, intellectual disability, oral apraxia, and epilepsy [41]. In the context of SB, its upregulation suggests a potential involvement in neural tube development. The association of *KCND3* with miRNAs miR-765 and miR-142-3p further highlights its regulatory significance. These findings could be valuable for prenatal diagnostics, as detecting *KCND3* and its related miRNAs through noninvasive screening methods, such as circulating fetal RNA in maternal blood, may enable early identification of SB. This could facilitate timely interventions and improved management of the condition [15]. The study by Aoulad Fares et al. explores how periconceptional interactions among maternal conditions, environmental factors, and genetic predispositions contribute to the pathogenesis and potential prevention of NTDs, such as SB.

**Figure 2:**
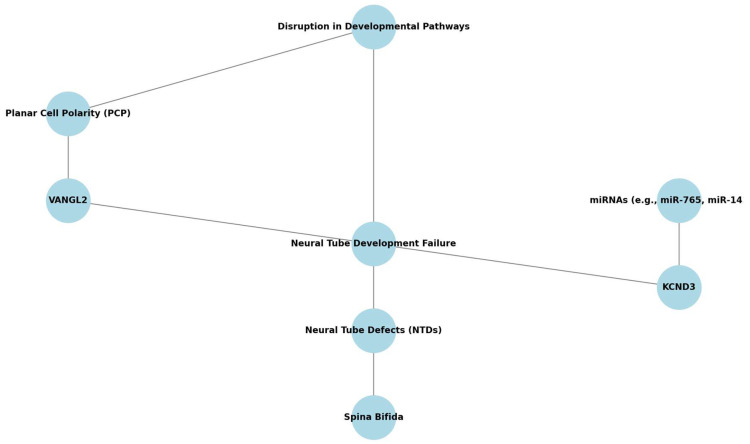
Genetic Pathways in Spina Bifida. This schematic highlights key genetic and epigenetic contributors to neural tube closure, including the planar cell polarity pathway (*VANGL2*), ion channel regulation (*KCND3*), and their regulation by microRNAs (*miR-765*, *miR-142-3p*). Environmental factors such as folate deficiency, oxidative stress, and telomere shortening are also included as upstream modulators. The integrative layout is designed to guide future research toward the most relevant mechanistic targets and regulatory interactions.

Figure 1 presents a conceptual framework integrating the genetic, epigenetic, and molecular disruptions implicated in the development of SB, considered within the broader category of NTDs. Disruptions in developmental pathways—such as planar cell polarity (PCP), folate metabolism, oxidative stress, and telomere maintenance—can impair neural tube closure, leading to NTDs. SB is highlighted as a clinical subtype of NTDs, emerging from these upstream failures. At the gene level, *VANGL2* is shown as a key component of the PCP pathway, whose mutations are associated with neural tube malformations. Concurrently, dysregulation of potassium channel gene *KCND3*—a known target of differentially expressed miRNAs such as miR-765 and miR-142-3p—may contribute to aberrant neurodevelopment in SB. These microRNAs act as epigenetic regulators that integrate environmental cues (e.g., folate deficiency, oxidative stress) with gene expression programs during early embryogenesis. The figure delineates these hierarchical and mechanistic relationships to illustrate how molecular perturbations may cascade into phenotypic outcomes such as SB. This integrative model facilitates a systems-level understanding of SB pathogenesis and highlights several molecular targets that may serve as diagnostic biomarkers or therapeutic entry points in future fetal interventions (Figure 2).

PCP—Planar Cell Polarity; NTDs—Neural Tube Defects; SB—Spina Bifida; miRNA—microRNA; KCND3—Potassium Voltage-Gated Channel Subfamily D Member 3; VANGL2—Van Gogh-Like Protein 2Recent advances in miRNA profiling have provided valuable insights into the molecular mechanisms underlying SB. In a preliminary study, distinct miRNA expression patterns were identified in maternal plasma and amniotic fluid samples from pregnancies affected by SB. Using NanoString technology, the expression levels of 798 miRNAs were evaluated in a cohort comprising six women carrying fetuses with SB and twenty women with euploid healthy fetuses. The study revealed significant downregulation of miRNAs, including miR-1253, miR-1290, miR-194-5p, miR-302d-3p, miR-3144-3p, miR-4536-5p, miR-548aa, miR-548ar-5p, miR-590-5p, miR-612, and miR-122-5p, while upregulation was observed in miR-320e, let-7b-5p, miR-23a-3p, miR-873-3p, and miR-30d-5p. These miRNAs were linked to critical pathways regulating stem cell pluripotency and other developmental processes. Notably, elevated levels of miR-320e in maternal plasma suggest its potential as a non-invasive biomarker for prenatal SB screening. This study highlights the importance of miRNA profiling in understanding the pathogenesis of SB and underscores the potential for developing miRNA-based diagnostic tools to improve prenatal detection and management of this congenital anomaly [17].

### 4.3. Folate Metabolism Associated Genes Analysis

The study conducted by Marini et al. investigates the genetic background of NTDs, particularly SB, despite the established benefits of folic acid supplementation. The study investigates rare alleles and genetic interactions in the folate metabolism pathway within a 480-member NTD case-control group. Using a pathway model to categorize genes, it seeks to identify complex genetic risk signatures. Ethnic variability is noted, with Hispanic individuals showing potential NTD risk related to purine biosynthesis, while non-Hispanic whites have risk associated with homocysteine metabolism. Consistent identification of a common, nonsynonymous risk allele (*MTHFD1* NM_005956.4:c.1958G > A; R653Q) in methylenetetrahydrofolate dehydrogenase, cyclohydrolase, and formyltetrahydrofolate synthetase 1 (*MTHFD1*) provides evidence linking purine synthesis to NTD risk. Additionally, a distinct genetic signature related to homocysteine metabolism was observed, specifically in non-Hispanic white individuals, significantly distinguishing NTD cases from controls. This research underscores the intricate nature of NTD risk, emphasizing the role of genetic interactions and ethnic variations in comprehending the underlying mechanisms [42]. Therefore, Price et al. explore the persistence of SB despite Canada’s folic acid fortification program, which has reduced NTD incidence. Their study examines whether NTDs can still occur in conditions of adequate folate due to disruptions in one-carbon metabolism and abnormal DNA methylation (DNAm). Using the Illumina Infinium HumanMethylation450 array, fetal tissue samples from various NTD status groups were analyzed for DNAm, and *MTHFR* genotypes were assessed. While no significant differences in DNAm of repetitive elements were found, the study identified differentially methylated sites in chorionic villi when comparing anencephaly to controls (4 sites) and in kidneys when comparing SB to controls (3342 sites). These findings indicate that the distinct DNAm patterns in SB kidneys may be a consequence of the neural tube defect itself or may reflect a common etiology between neural tube and renal abnormalities. Although minor DNAm changes were observed in other tissues, the data do not support the hypothesis of widespread genome-wide DNAm alterations in NTDs. This underscores the need to investigate alternative epigenetic and non-epigenetic mechanisms to gain a deeper understanding of the etiology of NTD, particularly in populations with adequate folate levels [43]. The studies listed in Table 2 were selected to illustrate representative examples of original research that contributed novel mechanistic insights into SB at the molecular or genetic level. Inclusion was based on methodological quality, relevance to one or more pathogenic pathways (e.g., folate metabolism, planar cell polarity, miRNA regulation), and translational significance. Only primary experimental studies published in peer-reviewed journals were included. Literature reviews, theoretical models, or hypothesis papers were excluded from this table to ensure focus on empirical findings. The selected studies are not intended as an exhaustive summary but as illustrative highlights aligned with the scope of this integrative review (Table 2).

### 4.4. Telomere Dynamics and SB: Unveiling the Genetic Link

One key biomarker analyzed is telomere length (TL). TLs consist of long nucleotide TTAGGG repeats and a protein complex at chromosome ends essential for maintaining chromosome integrity. Excessive shortening of TL, which indicates cellular senescence and genomic instability, is associated with an increased risk of age-related diseases. Additionally, TL shortening is linked to environmental and lifestyle factors that also contribute to NTD risk [44]. The study included observations from experiments with embryonic mice deficient in the telomerase gene, which demonstrated reduced TL and a failure of neural tube closure. These findings suggest that the neural tube development is particularly sensitive to TL loss and chromosomal instability [16]. A study by Zhao and Bai showed that maternal telomere length influences fetal telomere length [45]. Moreover, maternal health during pregnancy is a critical factor influencing telomere length programming in utero, potentially affecting the offspring’s health outcomes throughout their lifespan, including aspects of aging and longevity [46]. Several nutritional factors, including vitamins, minerals, and other bioactive dietary components, play a crucial role in mitigating the NTDs. These factors influence maternal health and fetal development, potentially impacting TL and overall pregnancy outcomes. Alternatively, it suggests that women with a higher risk of NTDs might already exhibit a more advanced biological age compared to their chronological age before pregnancy [16] (Table 2).

Therefore, due to the multifactorial pathomechanism underlying the occurrence of SB, which suggests an interplay between genetic predisposition and environmental factors, it is essential to investigate the hypothesis of whether TL may serve as a marker for SB itself or rather as an indicator specifically of environmental influences [47]. In a study by Goumy, the TL was found to be shortened in cases involving multiple congenital anomalies compared to fetuses with a single malformation, indicating a potential relationship between TL and the severity of the fetal phenotype. Consequently, measuring TL in amniocytes during pregnancy may serve as a novel predictive marker for pathological development [48].

Considering the TL evaluation methodology, the Southern blot-based telomere restriction fragment analysis has long been considered the gold standard for measuring TL in research settings. Despite its widespread use in laboratories over the years, this method has not been adopted in clinical diagnostics due to its labor-intensive nature, challenges in quantification, and the requirement for substantial quantities of high-quality DNA. Alternatively, relative telomere length can be assessed through quantitative PCR (qPCR), which is particularly well-suited for large-scale epidemiological studies [49]. Novel methods like high-throughput single telomere length analysis and telomere shortest length assay hold promise to potentially provide a more accurate assessment of telomere lengths [50].

Therapeutic approaches utilizing chemical activators of telomerase or telomerase-based gene therapy are currently under investigation in mouse models. These strategies aim to assess their potential for enhancing health, extending lifespan, and treating short telomere syndromes [51,52]. At present, there is a lack of literature data addressing prenatal telomere-related therapeutic approaches.

### 4.5. Protein Profiling

The findings of the study conducted by Thielen et al. focus on previously underexplored aspects of SB, specifically examining the consequences of cerebellar herniation and its associated metabolic alterations. This research utilizes a rat model of SB induced by retinoic acid to investigate the metabolic impact on the cerebellum during prenatal development. Through the analysis of this model at two specific time points, mid-late gestation (day 15) and term (day 20), in comparison to non-exposed and retinoic acid-exposed controls without MM, the results suggest the involvement of mechanisms related to oxidative stress and energy depletion in the affected neural tissue. These mechanisms are likely to contribute to further neural damage as the fetus continues to grow, and the compressed cerebellum undergoes development and herniation due to MM [53]. The study investigated the expression of serotonin receptors (5-HT) in the spinal cord and ganglia of human conceptuses aged 5 to 10 weeks, as well as in 9-week-old fetuses with SB. Utilizing immunohistochemistry and double immunofluorescence techniques, along with semi-quantitative and statistical analyses, the researchers assessed receptor expression and cell characteristics such as apoptosis and proliferation [54]. The findings revealed that during early neurulation, neuroblasts displayed moderate expression of SR1 and SR2, and mild expression of SR3. SR1 levels increased in motoneurons and sensory neurons, while SR2 and SR3 levels rose significantly in the floor and roof plates. In the ganglia, SR3 expression showed a marked increase during a specific developmental phase, whereas SR1 and SR2 continued to rise throughout the study period. In the malformed fetus with SB, there was reduced expression of SR1 and SR2, accompanied by increased SR3 expression, correlating with morphological abnormalities. Abnormalities in the roof plate were associated with an excess of apoptotic and proliferating cells, along with increased SR3 expression. Overall, the study highlights a unique serotonin receptor expression pattern in humans and underscores the importance of SR1 in neuronal differentiation, and SR2 and SR3 in regulating roof plate morphogenesis during both normal and disrupted development [54].

While the findings are novel, the clinical relevance of protein profiling in SB has not been fully established. Nevertheless, these results offer promising evidence of potential biomarkers and therapeutic targets in medicine, highlighting the need for further research.

## 5. Diaphragmatic Hernia

### 5.1. Etiology

**Figure 3:**
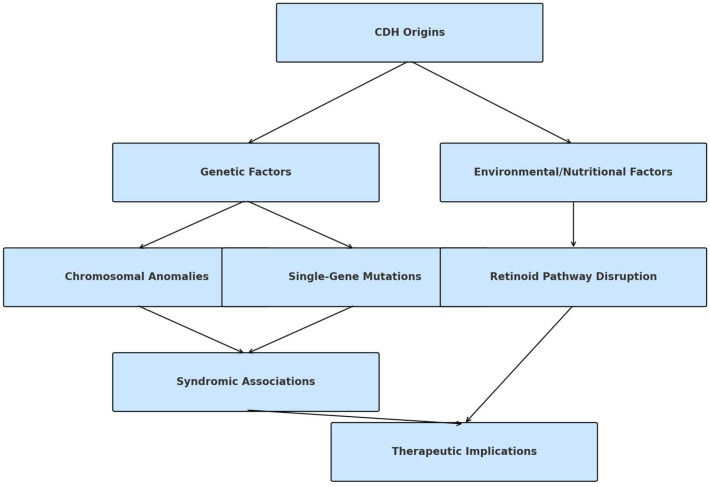
Simplified Pathogenetic Model of CDH. This diagram presents genetic and environmental factors contributing to abnormal diaphragm and lung development in CDH. Key genes include *GATA4*, *COUP-TFII*, and *FGFR2*, influenced by retinoic acid signaling, chromosomal deletions (e.g., 15q26), and teratogenic exposures. The figure underscores the interplay between developmental signaling and structural outcomes, serving as a framework for identifying candidate genes and pathways for future translational studies.

The exact origins of CDH remain unclear, but it generally results from a complex interplay of genetic factors, environmental exposures, including maternal medication use and toxin exposure, and nutritional deficiencies, particularly in folic acid and vitamin A [32]. CDH can manifest as an isolated anomaly or coexist with other organ system abnormalities. Various chromosomal anomalies, such as deletions and aneuploidies, as well as single-gene mutations (e.g., GATA binding protein 4 (*GATA4*) and low-density lipoprotein receptor-related protein 2 (*LRP2*), have been linked to CDH. Common aneuploidies associated with CDH include trisomy 18, trisomy 13, trisomy 21, and, less frequently, Turner syndrome (45, X). Specific syndromes associated with CDH include Pallister-Killian syndrome (12p tetrasomy); 8p23.1 deletion syndrome; Fryns syndrome, marked by multiple congenital defects and high perinatal mortality; and Cornelia de Lange syndrome, involving developmental delays and physical abnormalities due to the mutations in chromosome maintenance genes [33]. Notably, exposure to teratogenic substances during pregnancy, including mycophenolate mofetil, allopurinol, and lithium, has been reported in association with CDH [34]. Recent investigations also suggest the potential involvement of disruptions in the retinoid-signaling pathway as a contributing factor to CDH [55,56]. This signaling pathway influences mesodermal and neural crest cell migration, which are critical for proper diaphragm development. Expanding on therapeutic implications, targeting retinoid signaling pathways could provide potential avenues for correcting or mitigating diaphragmatic defects. Modulating retinoic acid levels or its receptor activity could improve diaphragm development in cases where retinoid signaling is disrupted. However, the therapeutic use of retinoids needs to be carefully controlled, as both excess and deficiency of retinoid signaling can lead to adverse effects, including teratogenic outcomes [57] (Figure 3).

### 5.2. Molecular Profiling

While mutations in individual genes have been linked to syndromic forms of CDH—such as *LRP2* mutations causing Donnai–Barrow syndrome, and mutations in *GATA4,* T-box transcription factor 4 (*TBX4*), and paired-like homeobox 2b (*PHOX2B*) contributing to other syndromic forms—only one case of apparently isolated CDH has been associated with a de novo mutation in the major gene *ZFPM2* zinc finger protein, FOG family member 2 (*FOG2*) [58,59,60,61]. Other genes implicated in syndromic CDH include NK2 homeobox 1 (*NKX2-1*), which is associated with thyroid dysgenesis and respiratory issues, and SRY-box transcription factor 9 (*SOX9*), which is involved in sex determination and cartilage formation [41,62]. The low sibling clustering of apparently isolated CDH cases also supports the role of de novo mutational events. However, these empirical observations do not rule out polygenic or multifactorial inheritance. Genes such as CRK-like proto-oncogene (*CRKL*) and myogenic factor 6 (*MYF6*) have been suggested in recent studies; however, formal evidence establishing a predominant inheritance pattern responsible for the majority of isolated CDH cases remains absent [62].

In 10–35% of cases of CDH, either whole or fragmented chromosome imbalances, extensive chromosome deletions/duplications, or intricate chromosome rearrangements can be detected through karyotyping. These abnormalities are most common in CDH cases that are not isolated and are diagnosed prenatally [63]. In an additional 3.5–13% of cases, which do not exhibit abnormalities in the karyotype, variations in the number of copies of specific DNA segments are observed [20]. These CNVs may encompass small deletions or duplications of genetic material and can be identified through chromosome microarray analysis, providing higher resolution compared to a standard karyotype [64]. Both aneuploidies and CNVs are linked to an increased risk of neonatal mortality [65].

In the research conducted by Scott et al., a genome-wide oligonucleotide-based array comparative genomic hybridization (aCGH) was employed in conjunction with real-time quantitative PCR analysis to detect, validate, and precisely locate chromosomal abnormalities in a cohort of 26 patients with CDH. The data suggest the presence of genes associated with CDH on chromosomes 2q37, 6p22-25, and 14q. Furthermore, the study narrows down the minimal deleted region for CDH on 15q26 to an interval containing COUP-TFII and only eight other known genes. While COUP-TFII is likely implicated in CDH development among patients with 15q26 deletions, no mutations in COUP-TFII were identified in 73 CDH samples. [64]. Furthermore, Robertson et al. conducted single-cell RNA sequencing with an unbiased clustering approach, revealing the existence of three distinct microvascular endothelial cell (mvEC) clusters. These clusters encompassed a general population of mvECs, a proliferative population, and a population characterized by high hemoglobin levels. Notably, only the mvEC cluster associated with CDH exhibited a unique transcriptomic signature related to inflammation when compared to the endothelial cells from healthy controls (2HC) and non-CDH (NC) cases. This distinctive CDH mvEC cluster displayed increased activation and adhesion of inflammatory cells and increased production of reactive oxygen species. Additionally, the CDH mvECs exhibited reduced gene expression of arbonic anhydrase 4 (*CA4*), apelin (*APLN*), and endothelin receptor type b (*EDNRB*), crucial markers for endothelial cells involved in lung development, gas exchange, and alveolar repair (mvCa4+). It was observed that mvCa4 + ECs were diminished in CDH cases (2HC [22.6%], NC [13.1%], and CDH [5.3%], *p* < 0.0001). In summary, these findings highlight the presence of transcriptionally distinct microvascular endothelial cell clusters in CDH, including the notably inflammatory mvEC cluster and the reduced population of mvCa4 + ECs, both of which may play a role in the disease’s pathogenesis. [66]. Dalmer et al. investigated genes linked to CDH and identified shared pathways contributing to abnormal diaphragm formation. They compiled a list of 218 CDH-associated genes from existing literature, categorized by CDH type, and performed gene ontology analysis to reveal enriched biological pathways. Findings demonstrate that pathways such as retinoic acid signaling in Bochdalek-type CDH, myogenesis in diaphragm eventration, and angiogenesis in central tendon defects are notably linked to various CDH clinical presentations, highlighting key genetic factors driving different CDH forms [67].

Furthermore, miRNAs, serving as epigenetic gene expression regulators, play a crucial role in fine-tuning various gene expression pathways. In the study conducted by Fabietti et al., an examination was carried out on the concentration of extracellular vesicles (EVs) and the expression of associated miRNAs in the amniotic and tracheal fluids of fetuses with CDH undergoing FETO [68]. Their research revealed that the concentration of EVs in these fluids was higher in infants who did not survive compared to those who did following FETO. Furthermore, the study provided insights into differential miRNA expression in this population. More specifically, in the pre-FETO amniotic fluid samples of non-surviving infants, there was an increased expression of miR-379-5p and miR-889-3p. The upregulation of miR-379-5p results in decreased expression of insulin-like growth factor 1 (*IGF1*), a key regulator of endothelin-1, which plays a role in vascular remodeling associated with pulmonary hypertension [69,70]. Additionally, miR-889-3p targets fibroblast growth factor receptor 2 (*FGFR2*), which is crucial for protecting and renewing epithelial cells and plays a pivotal role in various biological processes, including angiogenesis in pulmonary vasculature [71] (Table 3).

### 5.3. Protein Profiling

The study carried out by Bhutada et al. aimed to investigate proteomic changes in amniotic fluid associated with CDH, a severe birth defect often linked to pulmonary hypoplasia, pulmonary hypertension, and heart failure. The researchers collected amniotic fluid at term from women with normal pregnancies (n  =  5) and those carrying fetuses with CDH (n  =  5). After immuno-depletion of high-abundance proteins, they performed off-line fractionation and high-resolution tandem mass spectrometry, identifying 1036 proteins, with 218 showing differential abundance. Bioinformatics analysis revealed significant changes in GP6 signaling, the MSP–RON signaling in macrophages pathway, and networks associated with cardiovascular system development and function, connective tissue disorders, and dermatological conditions. Validation using ELISA in larger cohorts confirmed differences in selected proteins, including pulmonary surfactant protein B, osteopontin, kallikrein 5, and galectin-3. These proteins demonstrated statistically significant differences, providing potential diagnostic and predictive tools for CDH. The findings offer insights into the mechanisms underlying CDH and may contribute to improved clinical management [73]. In the following study performed by Wagner et al. aimed to elucidate the pathobiology underlying lung hypoplasia in CDH, a challenging birth defect associated with high mortality. Fetal rat lungs exposed to nitrofen in utero (CDH lungs) were compared with those exposed to the vehicle only (non-CDH control lungs) at the alveolar stage (E21).

Proteomic analysis identified a significant enrichment of inflammatory response proteins associated with cytokine signaling and Epstein-Barr virus in nitrofen-induced CDH lungs. Among the 218 significantly altered proteins were tenascin C, CREB-binding protein, tyrosine-protein kinase LYN, and signal transducer and activator of transcription 3 (STAT3). Notably, tenascin C was decreased around distal airway branches in nitrofen-exposed rat lungs and in human CDH lungs from stillborn fetuses. In contrast, STAT3 expression was markedly increased in the airway epithelium of nitrofen-exposed lungs at embryonic day 21 (E21). In an ex vivo model, inhibition of STAT3 following direct nitrofen exposure of fetal rat lung explants (E14.5) partially ameliorated the hypoplastic lung phenotype by promoting peripheral lung budding. Moreover, a significant increase in several cytokines associated with STAT3—including interleukin-15 (IL-15), interleukin-9 (IL-9), and interleukin-2 (IL-2)—was observed in fetal tracheal aspirates of CDH survivors compared to nonsurvivors following FETO. This unbiased proteomic analysis highlighted downstream inflammatory pathways likely implicated in the aberrant lung development characteristic of CDH, providing critical insights into the pathogenesis of this complex disorder and identifying potential targets for therapeutic intervention [74].

## 6. Discussion and Future Perspectives

The extremely low incidence of concurrent SB and CDH raises intriguing questions regarding the interplay of molecular pathways during embryogenesis. Although both anomalies may involve disruptions in folate metabolism, oxidative stress, and regulatory miRNAs (e.g., *miR-142-3p*, *miR-379-5p*), their pathogenic mechanisms remain largely distinct. SB arises from a failure of neural tube closure, driven primarily by planar cell polarity gene disruptions (e.g., *VANGL2*) and epigenetic alterations in neural precursors, while CDH is often associated with mesodermal defects affecting diaphragm and lung morphogenesis, involving retinoid signaling and genes such as *GATA4* and *COUP-TFII*. These distinct embryonic origins—neuroectoderm versus lateral plate mesoderm—as well as non-overlapping developmental time windows, likely account for the rarity of dual presentation. Nevertheless, shared upstream vulnerabilities may exist in genomic stability and the maternal environment, warranting further exploration into potential syndromic or polygenic mechanisms underlying co-occurrence. Thus, the examination of genetic factors in the context of SB and CDH provides crucial insights for future research and clinical applications. Despite their embryological divergence, SB and CDH may share upstream regulatory vulnerabilities, particularly involving oxidative stress response, disrupted folate-dependent methylation, and miRNA-mediated epigenetic control. For example, *miR-142-3p*, implicated in neural development, has also been detected in CDH-related placental expression profiles. These overlapping regulators may underlie occasional syndromic or dual presentations and offer targets for future cross-condition research. Moreover, the molecular candidates identified across studies reviewed—such as *VANGL2, KCND3, GATA4, LRP2*, and regulatory miRNAs including miR-765 and miR-142-3p—converge on a limited number of key developmental pathways [18,19,72]. These include the planar cell polarity (PCP) pathway, which is essential for coordinated cellular alignment during neural tube closure; the retinoid signaling pathway, which influences both neural and diaphragmatic tissue development; and ion channel regulation, which modulates early neurogenesis and tissue excitability [18,19,20,62]. While SB and CDH affect different embryonic structures and follow distinct developmental timelines, several environmental risk factors may intersect. Oxidative stress, maternal nutritional deficiencies (e.g., folate, vitamin A), and teratogenic exposures may act through common molecular mediators such as reactive oxygen species, DNA methylation changes, and non-coding RNAs. These shared influences may explain the rare co-occurrence of both anomalies and support the need for integrative maternal health strategies in early pregnancy. This convergence supports the hypothesis that disruptions in shared morphogenetic pathways—rather than condition-specific genes alone—may underlie multiple congenital anomalies. Accordingly, future research should further investigate these signaling axes as potential points of therapeutic convergence. These insights have direct clinical relevance: understanding the molecular drivers of SB and CDH facilitates earlier risk stratification through non-invasive biomarkers (e.g., circulating miRNAs such as *miR-320e* or *miR-379-5p*), supports the development of fetal therapeutic strategies (e.g., degradable tracheal occlusion devices, FGF-modulating treatments), and enables a shift toward personalized prenatal care. As research advances, such molecular tools may help physicians identify high-risk pregnancies earlier, tailor intervention timing, and potentially improve neonatal outcomes through targeted therapies.

Therefore, identifying and analyzing genes associated with these conditions enables the determination of miRNAs that regulate these genes, potentially leading to novel biomarkers for early detection. Additionally, understanding genetic profiles allows for the use of animal models to study the mechanisms underlying these anomalies, although much work remains to fully elucidate these processes. However, since protein concentrations reflect both maternal and fetal contributions, determining the specificity of these markers is challenging. This highlights the importance of genetic studies for developing precise diagnostic and therapeutic approaches. Future research should focus on advanced diagnostics, personalized medicine, and targeted interventions, while longitudinal studies and collaborative efforts are essential for improving healthcare outcomes for individuals affected by SB and CDH. This section highlights the importance of multidisciplinary collaboration, emphasizing the potential for geneticists and fetal surgeons to work together to translate research findings into clinical practice. For instance, the integration of genetic profiling with advanced surgical techniques could inform personalized treatment strategies. Furthermore, the exploration of advanced diagnostic tools, such as CRISPR and non-invasive prenatal testing (NIPT), holds promise for improving early detection and intervention in congenital anomalies like SB and CDH [75,76,77]. CRISPR, an acronym for clustered regularly interspaced short palindromic repeats and CRISPR-associated protein 9, is a powerful gene-editing tool capable of modifying the nucleotide sequence of genomes within living cells. This groundbreaking technology is accelerating the development and clinical translation of novel gene-editing therapies for genetic disorders [76].

### 6.1. In Vivo Models of SB

Galea et al. investigate the impact of mutations in the planar cell polarity gene *Vangl2*, which is linked to SB. While *Vangl2* mutations in mice have been shown to prevent the initiation of neural tube closure, this study focuses on *Vangl2′*s role in later neurulation stages. Using Grhl3Cre to delete *Vangl2* in the surface ectoderm and posterior neuropore (PNP) neuroepithelial cells, the researchers found that *Vangl2* disruption led to incomplete PNP closure after the ∼25-somite stage, resulting in caudal spina bifida in about 67% of embryos. The deletion affected cell body orientation in the dorsal surface ectoderm but not cell division direction. In the PNP, disruptions in *Vangl2* impair the mediolateral polarization of F-actin, leading to eversion of the caudal PNP and hindering the formation of Closure 5. Notably, in control embryos, the formation of Closure 5 is associated with a reduction in mechanical stress at the primary zippering point, as evidenced by the degree of neural fold separation observed following laser ablation of the zippering point. This mechanical stress adaptation did not occur in embryos with *Vangl2* disruption. Therefore, the disruption of *Vangl2*-dependent planar-polarized processes within the PNP neuroepithelium and SE obstructs the biomechanical adaptation at the zippering point, a crucial step associated with Closure 5 formation during the finalization of PNP closure. These findings illuminate the significance of *Vangl2* in the intricate processes of neural tube development and its implications for spina bifida. The mechanistic insights into *Vangl2* disruptions, such as impaired mediolateral polarization of F-actin and eversion of the caudal PNP, are compelling. However, their clinical relevance requires further exploration [78].

### 6.2. In Vivo Models of CDH: Targeting Novel Medical Approaches

The study conducted by Campiglio et al. explores alternatives to traditional balloon-based FETO for managing CDH. FETO is typically used to mitigate pulmonary hypoplasia by placing a balloon through a catheter to obstruct the trachea. However, this balloon must be removed after a certain period, which can lead to complications. To address these issues, the research aimed to develop an injectable, degradable tracheal plug that would not require prenatal removal. Two types of hydrogels were tested as potential substitutes: calcium-alginate and hyaluronan/methylcellulose blends (HA-MC). The study focused on optimizing the composition of these gels to meet design specifications for use as tracheal plugs. Key properties evaluated included gelation time, injectability, cohesiveness, sealing pressure, and persistence in anatomical tracheal models. In addition, the cytotoxicity and cell adhesion of mouse fibroblasts on the hydrogels were assessed to ensure biocompatibility. Both hydrogels demonstrated adjustable gelation times within the desired range. When injected into tracheal models, the hydrogels exhibited good cohesion and conformed well to anatomical shapes. Selected hydrogel compositions achieved effective sealing up to 80 cm H_2_O and maintained this seal for over four weeks. Swelling and weight loss of the hydrogels were influenced by their composition, varying from a few days to several weeks. Importantly, while neither hydrogel showed in vitro cytotoxicity, both exhibited low cell adhesion. The HA-MC blends, in particular, were noted for their shear-thinning behavior, making them a promising candidate for a single-surgery tracheal occlusion due to their adaptability and performance in the tracheal model [79]. This study explored alternatives to traditional balloon-based FETO for CDH management, focusing on calcium-alginate and HA-MC hydrogels. Comparative analysis of these hydrogels’ properties, such as gelation time, sealing capacity, and biocompatibility, reveals that HA-MC blends are particularly promising due to their shear-thinning behavior and adaptability in tracheal models. However, long-term safety and compatibility in complex in vivo models must be assessed to validate their clinical utility.

The following study, performed by Jesudason et al., investigates the impact of fibroblast growth factors (FGFs) and heparin on lung hypoplasia in the context of CDH using an organ culture model. Pulmonary hypoplasia, a severe reduction in lung growth, is a major contributor to the mortality associated with CDH. To explore potential therapeutic interventions, the researchers employed the nitrofen model, which induces lung hypoplasia in fetal rats. Pregnant Sprague-Dawley rats were administered nitrofen on day 9.5 of pregnancy to induce lung hypoplasia, while control rats received olive oil. On day 13.5 of gestation, normal and hypoplastic lung primordia were microdissected and cultured for up to 78 h in media supplemented with or without FGF-1, FGF-2, and/or heparin. The study assessed morphological development through measurements of terminal bud count, lung area, and lung perimeter. The results showed that normal lung tissue cultured with FGF-1 and heparin exhibited significant increases in area, perimeter, and bud count compared to control media. In contrast, in the nitrofen-induced hypoplastic lungs, FGF-1 and heparin treatment led to reduced lung development parameters. FGF-2 treatment resulted in increased lung area but reduced bud count and perimeter. Heparin alone did not produce substantial or sustained changes in lung morphology. The findings suggest an intrinsic abnormality in FGF processing in hypoplastic lungs induced by nitrofen. Heparin did not ameliorate the abnormal lung development. These results highlight the need for further research to understand the differential effects of FGFs and to explore mechanisms that could improve fetal lung growth in CDH [80]. This study investigated the differential effects of FGFs and heparin on lung development in nitrofen-induced hypoplastic lungs. While FGF-1 and heparin enhanced growth in normal lung tissue, they failed to improve hypoplastic lungs, suggesting intrinsic abnormalities in FGF signaling in these cases. Comparing the nitrofen model with other experimental systems could provide additional context, guiding future therapeutic approaches to improve lung development in CDH.

Peiro et al. explored fetal tracheal occlusion (TO) as an emerging therapy for improving lung growth and reducing mortality in CDH, even though respiratory challenges persist in survivors. Using a fetal sheep model of CDH with TO, the study conducted proteomic analysis on tracheal fluid, subsequently validating the findings in sheep lung tissue. The results showed that the proteomic profiles of CDH tracheal fluid were most similar to control lung, while CDH/TO lung was most similar to TO lung. Among 118 proteins altered in CDH, only 11 were reciprocally regulated in CDH/TO. The most significantly affected pathways and processes included cell proliferation, phosphatidylinositol 3-kinase/AKT/mammalian target of rapamycin signaling (PI3K/AKT/mTOR), inflammation, and microtubule dynamics. CDH suppressed cell proliferation and AKT-related signaling, while TO promoted these processes. Western blot analysis and immunohistochemistry revealed that epithelial PCNA and phosphorylated AKT were decreased in CDH and increased in TO and CDH/TO lungs. The WNT target Axin2 was decreased in the CDH lung without a significant increase in the CDH/TO lung. Cilia-related pathways were among the most dysregulated, with CDH lung showing an increase in acetylated α-tubulin and a relative increase in the number of ciliated cells. While TO was shown to improve lung growth and patient survival in CDH, the procedure substantially altered processes crucial for lung development and cell differentiation. Further understanding of these changes is deemed critical for enhancing lung health in infants with CDH treated with TO [81]. The finding that TO-modified lungs resemble TO-only lungs suggests that TO fundamentally shifts lung development toward a unique trajectory. This shift highlights the need for refinement of TO techniques to minimize adverse effects. Proteomic analyses reveal key dysregulated pathways, such as PI3K/AKT/mTOR signaling and cilia-related processes. Targeting specific dysregulated pathways with pharmacological agents could mitigate these developmental changes, optimizing TO outcomes for infants with CDH.

### 6.3. Targeting Novel Medical Approaches

The work by Campiglio et al. highlights innovations in injectable, degradable tracheal plugs as alternatives to balloon-based FETO. These plugs offer the potential for a single-surgery solution, addressing complications associated with prenatal balloon removal. Meanwhile, studies by Jesudason et al. and Peiro et al. underscore the complex interplay of growth factors and signaling pathways in lung development. Together, these findings emphasize the importance of integrating proteomics, advanced biomaterials, and molecular therapeutics to advance the management of SB and CDH [79,80,81].

## 7. Conclusions

Exploring mechanistic overlaps between distinct congenital anomalies like SB and CDH may inform more holistic prenatal screening approaches and reveal shared modifiable risk factors. Insights into key genetic contributors, such as *KCND3* and *VANGL2* in SB and COUP-TFII on chromosome 15q26 in CDH, have advanced our understanding of the molecular mechanisms underlying these conditions. Furthermore, metabolic disruptions like telomere shortening and folate metabolism defects, alongside microRNAs influencing gene expression, underscore the multifactorial nature of SB and CDH. Innovative therapeutic approaches, including degradable tracheal plugs and growth factor-based treatments, offer promising avenues for improving outcomes in CDH. Similarly, in SB, genetic and in vitro studies continue to shed light on neural tube development and its associated disruptions, paving the way for targeted prenatal interventions. These findings emphasize the critical importance of integrating genetic research with advanced diagnostic tools, such as NIPT, and leveraging novel therapeutic strategies to enhance both fetal and neonatal care. By bridging genetic insights with clinical practice, these advancements hold the potential to redefine the management of congenital anomalies, fostering a collaborative, multidisciplinary approach to fetal medicine and pediatric healthcare. Finally, while SB and CDH are generally studied as separate entities, this review underscores the importance of integrative approaches in understanding rare but biologically plausible dual presentations. The convergence of certain molecular risk factors—such as oxidative damage, telomere shortening, and miRNA dysregulation—suggests that cross-system mechanisms may occasionally disrupt both neural and mesodermal development. The integration of genetic and epigenetic biomarkers into prenatal screening protocols, combined with the refinement of fetal therapies guided by molecular targets, represents a promising direction for personalized fetal medicine. Understanding how environmental triggers modulate shared molecular pathways in early development may offer preventive opportunities applicable across multiple congenital disorders. Thus, this review serves as a foundation for translating mechanistic discoveries into practical tools for risk prediction, diagnosis, and intervention.

## 8. Key Take-Home Messages

- Shared mechanisms, distinct outcomes. While SB and CDH rarely co-occur, both are influenced by folate metabolism, oxidative stress, and miRNA-mediated regulation.
- Top gene–pathway candidates. *VANGL2*, *KCND3* (SB), and *GATA4*, *COUP-TFII* (CDH) emerge as high-priority molecular targets for future functional studies.
- miRNAs as non-invasive biomarkers. Dysregulated circulating miRNAs (e.g., *miR-320e* in SB; *miR-379-5p* in CDH) show promise for early screening.
- Therapeutic horizons. Novel prenatal interventions—fetoscopic repair (SB), degradable tracheal plugs, and growth-factor modulation (CDH)—illustrate how mechanistic insights can drive clinical innovation.
- Research gaps. Systems-level studies that integrate multi-omics data with in vivo models are needed to unravel why dual SB + CDH presentations are exceptionally rare.

## Figures and Tables

**Table 1:** Prevalence and Classifications of SB and CDH.

Condition	Prevalence	Classification	Key Characteristics	References
**SB**	Global incidence: 1.0–10.0 per 1000 newborns Approx. 1427 cases annually (1 in 2758 live births)	NTDs: Anencephaly: Incomplete closure in the cranial region SB: Incomplete closure below the cranial region SB Subtypes: Myelomeningocele: >90% of cases Meningocele - Lipomeningocele	Myelomeningocele: Protrusion of nervous tissue and protective coverings through a vertebral defect Most common type of SB	[1,35,36]
**CDH**	Global incidence: 1–4 per 10,000 live births	Types of Hernias: - Bochdalek Hernia: 70–75%; postero-lateral defect (mainly left side) - Morgagni Hernia: 20–25%; anteromedial defect - Central Hernia: 2–5%; rare and severe	- Bochdalek Hernia: Most prevalent, affecting diaphragm development - Central Hernia: Rare, associated with grave prognosis	[11,32,34,37]

NTD: Neural Tube Defects; SB: Spina Bifida; CDH: Congenital Diaphragmatic Hernia.

**Table 2:** Summary of Key Findings and Methodologies in Recent Research on Genetic and Molecular Aspects of Spina Bifida.

Aspect/Source	[15]	[16]	[17,42]
Objective	Analyze transcription profiles from human amniocytes to identify genes related to SB and healthy controls.	Investigate how periconceptional factors affect NTD pathogenesis and prevention.	Examine the persistence of SB in a folate-replete population and explore potential epigenetic factors.
Data Source	Gene Expression Omnibus database	Experiments with embryonic mice and literature review	Fetal tissue samples collected from different NTD status groups in Canada
Initial Data Processing	Outlier data identified and removed using PCA and sample clustering.	N/A	Analysis of DNA methylation (DNAm) using the Illumina Infinium HumanMethylation450 array.
Analysis Method	WGCNA	Telomere length analysis and experimental observations	Assessment of DNAm in chorionic villi and kidney samples, and evaluation of Methylenetetrahydrofolate reductase genotypes.
Dataset Analyzed	GSE4182 dataset	Observations from embryonic mice deficient in the telomerase gene	Fetal tissue DNAm data and Methylenetetrahydrofolate reductase genotypes.
Genes Analyzed	5407 genes categorized into 19 distinct modules	Telomere length associated with developmental processes	Differentially methylated sites in anencephaly and spina bifida compared to controls.
Findings	Identified 967 candidate genes associated with SB; miRNA–mRNA network with 4 miRNAs and 39 mRNAs.	Excessive TL shortening linked to neural tube closure failure in mice.	No significant DNAm differences in repetitive elements; specific DNAm changes observed in chorionic villi and kidneys.
Key Genes/MiRNAs Identified	KCND3 (upregulated in SB); associated miRNAs: miR-765 and miR-142-3p.	N/A	Notable DNAm changes in spina bifida kidneys and anencephaly.
Validation Method	Quantitative real-time PCR (qRT-PCR)	N/A	Analysis of DNAm and genotyping results.
Hypothesis/Conclusions	KCND3 and its associated miRNAs are promising for early detection and noninvasive screening in SB.	Long-term exposure to harmful factors accelerates maternal aging, increasing NTD risk. Alternatively, higher NTD risk may correlate with advanced biological age before pregnancy.	Persistent NTDs in folate-sufficient populations may involve alternative epigenetic mechanisms, with specific DNAm changes observed in spina bifida kidneys.

N/A: not applicable; NTD: Neural Tube Defects; SB: Spina Bifida; PCA: Principal Component Analysis; WGCNA: Weighted Gene Co-expression Network Analysis; TL: Telomere Length; miRNA: MicroRNA; qRT-PCR: Quantitative Real-Time Polymerase Chain Reaction; DNAm: DNA Methylation.

**Table 3:** Summary of Key Findings and Methodologies in Recent Research on Genetic and Molecular Aspects of Congenital Diaphragmatic Hernia.

Aspect/Source	[64]	[66]	[67,72]	[68]
Objective	Detect and validate chromosomal abnormalities in CDH patients.	Identify transcriptomic signatures in microvascular endothelial cells (mvECs) related to CDH.	Investigate genes associated with CDH and their pathways.	Examine extracellular vesicle (EV) concentrations and miRNA expression in CDH fetuses.
Data Source	26 patients with CDH	Single-cell RNA sequencing of mvECs from CDH and control samples	Literature review and gene ontology analysis	Amniotic and tracheal fluids of fetuses undergoing FETO
Analysis Method	Genome-wide oligonucleotide-based array comparative genomic hybridization (aCGH) and quantitative real-time PCR.	Unbiased clustering approach in single-cell RNA sequencing.	Gene ontology analysis and review of existing literature on CDH-associated genes.	Analysis of EV concentrations and miRNA expression levels.
Findings	Identified genes on chromosomes 2q37, 6p22-25, and 14q; minimal deleted region on 15q26 includes COUP-TFII.	Unique inflammatory transcriptomic signature in CDH-associated mvECs; reduced mvCa4 + ECs in CDH cases.	218 genes associated with CDH identified; distinct pathways related to various CDH types.	Increased EV concentration in non-surviving CDH infants; upregulation of miR-379-5p and miR-889-3p in pre-FETO amniotic fluid.
Key Genes/MiRNAs Identified	COUP-TFII and genes on chromosomes 2q37, 6p22-25, 14q; 15q26 deletions.	miR-379-5p, miR-889-3p; inflammatory markers in mvECs.	218 CDH-associated genes; pathways related to retinoic acid signaling, myogenesis, and angiogenesis.	miR-379-5p targets IGF1; miR-889-3p targets FGFR2.
Validation Method	Comparison of chromosomal abnormalities using aCGH and PCR.	Comparison of transcriptomic profiles between CDH and control endothelial cells.	Gene ontology analysis and comparison of gene functions.	Analysis of miRNA expression in pre-FETO amniotic fluid and association with survival outcomes.
Hypothesis/Conclusions	Oligonucleotide-based aCGH and PCR are effective for identifying and mapping CDH-related chromosomal changes.	Distinct mvEC clusters in CDH have unique inflammatory signatures and reduced mvCa4 + ECs.	Gene pathways are significantly associated with different forms of CDH, providing insights into disease mechanisms.	Elevated EV concentrations and specific miRNAs in CDH fetuses are linked to survival outcomes and disease mechanisms.

CDH: Congenital Diaphragmatic Hernia; aCGH: Array Comparative Genomic Hybridization; PCR: Polymerase Chain Reaction; mvEC: Microvascular Endothelial Cells; miRNA: MicroRNA; EV: Extracellular Vesicles; FETO: Fetal Endoscopic Tracheal Occlusion; IGF1: Insulin-like Growth Factor 1; FGFR2: Fibroblast Growth Factor Receptor 2.

## Data Availability

Not applicable.

## References

[1] Mitchell L.E., Adzick N.S., Melchionne J., Pasquariello P.S., Sutton L.N., Whitehead A.S. (2004). Spina bifida. Lancet.

[2] Chandrasekharan P.K., Rawat M., Madappa R., Rothstein D.H., Lakshminrusimha S. (2017). Congenital Diaphragmatic hernia—A review. Matern. Health Neonatol. Perinatol..

[3] Rivas J.F.G., Clugston R.D. (2023). The etiology of congenital diaphragmatic hernia: The retinoid hypothesis 20 years later. Pediatr. Res..

[4] Paoletti M., Raffler G., Gaffi M.S., Antounians L., Lauriti G., Zani A. (2020). Prevalence and risk factors for congenital diaphragmatic hernia: A global view. J. Pediatr. Surg..

[5] Shookhoff J., Gallicano G.I. (2010). A new perspective on neural tube defects: Folic acid and microRNA misexpression. Genesis.

[6] Seidahmed M.Z., Abdelbasit O.B., Shaheed M.M., Alhussein K.A., Miqdad A.M., Samadi A.S., Khalil M.I., Al-Mardawi E., Salih M.A. (2014). Genetic, chromosomal, and syndromic causes of neural tube defects. Saudi Med. J..

[7] Mohd-Zin S.W., Marwan A.I., Chaar M.K.A., Ahmad-Annuar A., Abdul-Aziz N.M. (2017). Spina Bifida: Pathogenesis, Mechanisms, and Genes in Mice and Humans. Scientifica.

[8] Perveen S., Frigeni M., Benveniste H., Kurepa D. (2022). Cellular, molecular, and metabolic aspects of developing lungs in congenital diaphragmatic hernia. Front. Pediatr..

[9] Mukherjee S., Pasulka J. (2017). Care for Adults with Spina Bifida: Current State and Future Directions. Top. Spinal Cord Inj. Rehabil..

[10] Thompson L.P., Al-Hasan Y. (2012). Impact of Oxidative Stress in Fetal Programming. J. Pregnancy.

[11] Prasad R., Prasad R. (2020). Congenital Diaphragmatic Hernia: A Major Challenge for Neonatologists. Congenital Anomalies in Newborn Infants—Clinical and Etiopathological Perspectives.

[12] Joyeux L., Chalouhi G., Ville Y., Sapin E. (2014). Maternal-fetal surgery for spina bifida: Future perspectives. J. Gynecol. Obstet. Biol. Reprod..

[13] Renik-Jankowska W., Buczyńska A., Sidorkiewicz I., Kosiński P., Zbucka-Krętowska M. (2024). Exploring new perspectives on congenital diaphragmatic hernia: A comprehensive review. Biochim. Biophys. Acta Mol. Basis. Dis..

[14] Crider K.S., Qi Y.P., Yeung L.F., Mai C.T., Zauche L.H., Wang A., Daniels K., Williams J.L. (2022). Folic Acid and the Prevention of Birth Defects: 30 Years of Opportunity and Controversies. Annu. Rev. Nutr..

[15] Li Z., Feng J., Yuan Z. (2020). Key Modules and Hub Genes Identified by Coexpression Network Analysis for Revealing Novel Biomarkers for Spina Bifida. Front. Genet..

[16] Fares D.A., Schalekamp-Timmermans S., Nawrot T.S., Steegers-Theunissen R.P.M. (2020). Preconception telomere length as a novel maternal biomarker to assess the risk of spina bifida in the offspring. Birth Defects Res..

[17] Buczyńska A., Sidorkiewicz I., Niemira M., Krętowski A.J., Węgrzyn P., Kosiński P., Zbucka-Krętowska M. (2024). Identification of MicroRNA Profiles in Fetal Spina Bifida: The Role in Pathomechanism and Diagnostic Significance. Int. J. Mol. Sci..

[18] Wolujewicz P., Aguiar-Pulido V., AbdelAleem A., Nair V., Thareja G., Suhre K., Shaw G.M., Finnell R.H., Elemento O., Ross M.E. (2021). Genome-wide investigation identifies a rare copy-number variant burden associated with human spina bifida. Genet. Med..

[19] Scott D.A., Gofin Y., Berry A.M., Adams A.D. (2022). Underlying Genetic Etiologies of Congenital Diaphragmatic Hernia. Prenat. Diagn..

[20] Yu L., Wynn J., Ma L., Guha S., Mychaliska G.B., Crombleholme T.M., Azarow K.S., Lim F.Y., Chung D.H., Potoka D. (2012). De novo copy number variants are associated with congenital diaphragmatic hernia. J. Med. Genet..

[21] Zhu Q., High F.A., Zhang C., Cerveira E., Russell M.K., Longoni M., Joy M.P., Ryan M., Mil-Homens A., Bellfy L. (2018). Systematic analysis of copy number variation associated with congenital diaphragmatic hernia. Proc. Natl. Acad. Sci. USA.

[22] Khawale R., Kanetkar S.R., Patil M. (2024). Impact of Hypothyroidism in Pregnancy on Feto-Maternal Outcomes: A Prospective Observational Study. Cureus.

[23] Miller J.L., Groves M.L., Baschat A.A. (2019). Fetoscopic spina bifida repair. Minerva Obstet. Gynecol..

[24] Doné E., Gratacos E., Nicolaides K.H., Allegaert K., Valencia C., Castañon M., Martinez J., Jani J., Van Mieghem T., Greenough A. (2013). Predictors of neonatal morbidity in fetuses with severe isolated congenital diaphragmatic hernia undergoing fetoscopic tracheal occlusion. Ultrasound Obstet. Gynecol..

[25] Moher D., Liberati A., Tetzlaff J., Altman D.G., Altman D., Antes G., Atkins D., Barbour V., Barrowman N., Berlin J.A. (2009). Preferred reporting items for systematic reviews and meta-analyses: The PRISMA statement. PLoS Med..

[26] Hutton B., Salanti G., Caldwell D.M., Chaimani A., Schmid C.H., Cameron C., Ioannidis J.P.A., Straus S., Thorlund K., Jansen J.P. (2015). The PRISMA extension statement for reporting of systematic reviews incorporating network meta-analyses of health care interventions: Checklist and explanations. Ann. Intern. Med..

[27] Stang A., Jonas S., Poole C. (2018). Case study in major quotation errors: A critical commentary on the Newcastle–Ottawa scale. Eur. J. Epidemiol..

[28] Atta C.A.M., Fiest K.M., Frolkis A.D., Jette N., Pringsheim T., Germaine-Smith C.S., Rajapakse T., Kaplan G.G., Metcalfe A. (2016). Global birth prevalence of spina bifida by folic acid fortification status: A systematic review and meta-analysis. Am. J. Public Health.

[29] Au K.S., Ashley-Koch A., Northrup H. (2010). Epidemiologic and genetic aspects of spina bifida and other neural tube defects. Dev. Disabil. Res. Rev..

[30] Mai C.T., Isenburg J.L., Canfield M.A., Meyer R.E., Correa A., Alverson C.J., Lupo P.J., Riehle-Colarusso T., Cho S.J., Aggarwal D. (2019). National population-based estimates for major birth defects, 2010–2014. Birth Defects Res..

[31] Sahmat A., Gunasekaran R., Mohd-Zin S.W., Balachandran L., Thong M.-K., Engkasan J.P., Ganesan D., Omar Z., Azizi A.B., Ahmad-Annuar A. (2017). The prevalence and distribution of spina bifida in a single major referral center in Malaysia. Front. Pediatr..

[32] Dumpa V., Chandrasekharan P. (2023). Congenital Diaphragmatic Hernia. StatPearls.

[33] Kraemer U.S., Leeuwen L., Krasemann T.B., Wijnen R.M.H., Tibboel D., Ijsselstijn H. (2018). Characteristics of Infants With Congenital Diaphragmatic Hernia Who Need Follow-Up of Pulmonary Hypertension. Pediatr. Crit. Care Med..

[34] Keijzer R., Puri P. (2010). Congenital diaphragmatic hernia. Semin. Pediatr. Surg..

[35] Copp A.J., Adzick N.S., Chitty L.S., Fletcher J.M., Holmbeck G.N., Shaw G.M. (2015). Spina bifida. Nat. Rev. Dis. Prim..

[36] Brea C.M., Munakomi S. (2023). Spina Bifida. StatPearls.

[37] Harting M.T. (2017). Congenital diaphragmatic hernia-associated pulmonary hypertension. Semin. Pediatr. Surg..

[38] Adzick N.S. (2015). Prenatal diagnosis and treatment of spina bifida. Fetal Diagn. Ther..

[39] Tinker S.C., Devine O., Mai C., Hamner H.C., Reefhuis J., Gilboa S.M., Dowling N.F., Honein M.A. (2013). Estimate of the potential impact of folic acid fortification of corn masa flour on the prevention of neural tube defects. Birth Defects Res. Part A: Clin. Mol. Teratol..

[40] Buczyńska A., Sidorkiewicz I., Hameed A., Krętowski A.J., Zbucka-Krętowska M. (2022). Future Perspectives in Oxidative Stress in Trisomy 13 and 18 Evaluation. J. Clin. Med..

[41] Smets K., Duarri A., Deconinck T., Ceulemans B., van de Warrenburg B.P., Züchner S., Gonzalez M.A., Schüle R., Synofzik M., Van der Aa N. (2015). First de novo KCND3 mutation causes severe Kv4.3 channel dysfunction leading to early onset cerebellar ataxia, intellectual disability, oral apraxia and epilepsy. BMC Med. Genet..

[42] Marini N.J., Hoffmann T.J., Lammer E.J., Hardin J., Lazaruk K., Stein J.B., Gilbert D.A., Wright C., Lipzen A., Pennacchio L.A. (2011). A Genetic Signature of Spina Bifida Risk from Pathway-Informed Comprehensive Gene-Variant Analysis. PLoS ONE.

[43] Price E.M., Peñaherrera M.S., Portales-Casamar E., Pavlidis P., Van Allen M.I., McFadden D.E., Robinson W.P. (2016). Profiling placental and fetal DNA methylation in human neural tube defects. Epigenetics Chromatin.

[44] Scholten R.H., Møller P., Andersen Z.J., Dehlendorff C., Khan J., Brandt J., Ketzel M., Knudsen L.E., Mathiesen L. (2021). Telomere length in newborns is associated with exposure to low levels of air pollution during pregnancy. Environ. Int..

[45] Zhao X.-X., Le Bai L. (2024). Correlation between telomere shortening in maternal peripheral blood and fetal aneuploidy. BMC Pregnancy Childbirth.

[46] Chen L., Tan K.M.L., Gong M., Chong M.F.F., Tan K.H., Chong Y.S., Meaney M.J., Gluckman P.D., Eriksson J.G., Karnani N. (2022). Variability in newborn telomere length is explained by inheritance and intrauterine environment. BMC Med..

[47] Factor-Litvak P., Susser E., Aviv A. (2017). Environmental Exposures, Telomere Length at Birth, and Disease Susceptibility in Later Life. JAMA Pediatr..

[48] Hemann M.T., Strong M.A., Hao L.-Y., Greider C.W. (2001). The shortest telomere, not average telomere length, is critical for cell viability and chromosome stability. Cell.

[49] Alder J.K., Hanumanthu V.S., Strong M.A., DeZern A.E., Stanley S.E., Takemoto C.M., Danilova L., Applegate C.D., Bolton S.G., Mohr D.W. (2018). Diagnostic utility of telomere length testing in a hospital-based setting. Proc. Natl. Acad. Sci. USA.

[50] Bhala S., Savage S.A. (2023). What is the future of telomere length testing in telomere biology disorders?. Expert Rev. Hematol..

[51] Bär C., Blasco M.A. (2016). Telomeres and telomerase as therapeutic targets to prevent and treat age-related diseases. F1000Research.

[52] Martínez P., Blasco M.A. (2017). Telomere-driven diseases and telomere-targeting therapies. J. Cell Biol..

[53] Thielen E., Oria M., Watanabe-Chailland M., Lampe K., Romick-Rosendale L., Peiro J.L. (2023). Non-Targeted Metabolic Profiling of Cerebellum in Spina Bifida Fetal Rats. Metabolites.

[54] Punda H., Mardesic S., Filipovic N., Kosovic I., Benzon B., Ogorevc M., Bocina I., Kolic K., Vukojevic K., Saraga-Babic M. (2021). Expression pattern of 5-ht (Serotonin) receptors during normal development of the human spinal cord and ganglia and in fetus with cervical spina bifida. Int. J. Mol. Sci..

[55] Clugston R.D., Zhang W., Álvarez S., de Lera A.R., Greer J.J. (2010). Understanding abnormal retinoid signaling as a causative mechanism in congenital diaphragmatic hernia. Am. J. Respir. Cell Mol. Biol..

[56] Coste K., Beurskens L.W.J.E., Blanc P., Gallot D., Delabaere A., Blanchon L., Tibboel D., Labbé A., Rottier R.J., Sapin V. (2015). Metabolic disturbances of the vitamin A pathway in human diaphragmatic hernia. Am. J. Physiol. Lung Cell. Mol. Physiol..

[57] Friedmacher F., Puri P. (2024). Disruptions in retinoic acid signaling pathway contribute to abnormal lung development in congenital diaphragmatic hernia: A therapeutic potential for retinoids to attenuate pulmonary hypoplasia. Pediatr. Res..

[58] Ackerman K.G., Herron B.J., O Vargas S., Huang H., Tevosian S.G., Kochilas L., Rao C., Pober B.R., Babiuk R.P., A Epstein J. (2005). Fog2 Is Required for Normal Diaphragm and Lung Development in Mice and Humans. PLoS Genet..

[59] Yu L., Wynn J., Cheung Y.H., Shen Y., Mychaliska G.B., Crombleholme T.M., Azarow K.S., Lim F.Y., Chung D.H., Potoka D. (2013). Variants in GATA4 are a rare cause of familial and sporadic congenital diaphragmatic hernia. Hum. Genet..

[60] Fernández R.M., Mathieu Y., Luzón-Toro B., Núñez-Torres R., González-Meneses A., Antiñolo G., Amiel J., Borrego S., Veitia R.A. (2013). Contributions of PHOX2B in the Pathogenesis of Hirschsprung Disease. PLoS ONE.

[61] Kantarci S., Ragge N.K., Thomas N.S., Robinson D.O., Noonan K.M., Russell M.K., Donnai D., Raymond F.L., Walsh C.A., Donahoe P.K. (2008). Donnai–Barrow syndrome (DBS/FOAR) in a child with a homozygous *LRP2* mutation due to complete chromosome 2 paternal isodisomy. Am. J. Med. Genet. Part A.

[62] Pober B. (2008). Genetic aspects of human congenital diaphragmatic hernia. Clin. Genet..

[63] Zaiss I., Kehl S., Link K., Neff W., Schaible T., Sütterlin M., Siemer J. (2011). Associated malformations in congenital diaphragmatic hernia. Am. J. Perinatol..

[64] Scott D.A., Klaassens M., Holder A.M., Lally K.P., Fernandes C.J., Galjaard R.-J., Tibboel D., de Klein A., Lee B. (2007). Genome-wide oligonucleotide-based array comparative genome hybridization analysis of non-isolated congenital diaphragmatic hernia. Hum. Mol. Genet..

[65] Wynn J., Yu L., Chung W.K. (2014). Genetic causes of congenital diaphragmatic hernia. Semin. Fetal Neonatal Med..

[66] Robertson J.O., Bazeley P., Erzurum S.C., Asosingh K. (2023). Single-cell transcriptomic profiling of microvascular endothelial cell heterogeneity in congenital diaphragmatic hernia. Sci. Rep..

[67] Dalmer T.R.A., Clugston R.D. (2018). Gene ontology enrichment analysis of congenital diaphragmatic hernia-associated genes. Pediatr. Res..

[68] Fabietti I., Nardi T., Favero C., Dioni L., Cantone L., Pergoli L., Hoxha M., Pinatel E., Mosca F., Bollati V. (2021). Extracellular vesicles and their mirna content in amniotic and tracheal fluids of fetuses with severe congenital diaphragmatic hernia undergoing fetal intervention. Cells.

[69] Tian L., Zhang L., Liu J., Guo T., Gao C., Ni J. (2014). Effects of TSH on the function of human umbilical vein endothelial cells. J. Mol. Endocrinol..

[70] He X., Zhao L., Yue L., Zhang W., Wang W., Fu Y., Feng Y., Fu F. (2019). The relationship between IGF1 and the expression spectrum of miRNA in the placenta of preeclampsia patients. Ginekol. Pol..

[71] Zhu Q., Li Y., Li L., Guo M., Zou C., Xu Y., Yang Z. (2021). MicroRNA-889-3p restrains the proliferation and epithelial—Mesenchymal transformation of lung cancer cells via down-regulation of Homeodomain-interacting protein kinase 1. Bioengineered.

[72] Srisupundit K., Brady P.D., Devriendt K., Fryns J., Cruz-Martinez R., Gratacos E., Deprest J.A., Vermeesch J.R. (2010). Targeted array comparative genomic hybridisation (array CGH) identifies genomic imbalances associated with isolated congenital diaphragmatic hernia (CDH). Prenat. Diagn..

[73] Bhutada S., Tran-Lundmark K., Kramer B., Conner P., Lowry A.M., Blackstone E., Frenckner B., Mesas-Burgos C., Apte S.S. (2023). Identification of protein biomarkers associated with congenital diaphragmatic hernia in human amniotic fluid. Sci. Rep..

[74] Wagner R., Lieckfeldt P., Piyadasa H., Markel M., Riedel J., Stefanovici C., Peukert N., Patel D., Derraugh G., Min S.A.L. (2023). Proteomic Profiling of Hypoplastic Lungs Suggests an Underlying Inflammatory Response in the Pathogenesis of Abnormal Lung Development in Congenital Diaphragmatic Hernia. Ann. Surg..

[75] Jayashankar S.S., Nasaruddin M.L., Hassan M.F., Dasrilsyah R.A., Shafiee M.N., Ismail N.A.S., Alias E. (2023). Non-Invasive Prenatal Testing (NIPT): Reliability, Challenges, and Future Directions. Diagnostics.

[76] Demirci S., Leonard A., Essawi K., Tisdale J.F. (2021). CRISPR-Cas9 to induce fetal hemoglobin for the treatment of sickle cell disease. Mol. Ther. Methods Clin. Dev..

[77] Papizan J.B., Porter S.N., Sharma A., Pruett-Miller S.M. (2020). Therapeutic gene editing strategies using CRISPR-Cas9 for the β-hemoglobinopathies. J. Biomed. Res..

[78] Galea G.L., Nychyk O., Mole M.A., Moulding D., Savery D., Nikolopoulou E., Henderson D.J., Greene N.D.E., Copp A.J. (2018). Vangl2 disruption alters the biomechanics of late spinal neurulation leading to spina bifida in mouse embryos. Dis. Model. Mech..

[79] Campiglio C.E., Villonio M., Dellacà R.L., Mosca F., Draghi L. (2019). An injectable, degradable hydrogel plug for tracheal occlusion in congenital diaphragmatic hernia (CDH). Mater. Sci. Eng. C Mater. Biol. Appl..

[80] Jesudason E.C., Connell M., Fernig D.G., Lloyd D.A., Losty P.D. (2000). In vitro effects of growth factors on lung hypoplasia in a model of congenital diaphragmatic hernia. J. Pediatr. Surg..

[81] Peiro J.L., Oria M., Aydin E., Joshi R., Cabanas N., Schmidt R., Schroeder C., Marotta M., Varisco B.M. (2018). Proteomic profiling of tracheal fluid in an ovine model of congenital diaphragmatic hernia and fetal tracheal occlusion. Am. J. Physiol. Lung Cell. Mol. Physiol..

